# Key lime (*Citrus aurantifolia*) inhibits the growth of triple drug resistant *Helicobacter pylori*

**DOI:** 10.1186/s13099-018-0244-y

**Published:** 2018-05-21

**Authors:** Su-Mi Lee, Seon-Young Park, Moon-Ju Kim, Eun-Ae Cho, Chung-Hwan Jun, Chang-Hwan Park, Hyun-Soo Kim, Sung-Kyu Choi, Jong-Sun Rew

**Affiliations:** Department of Internal Medicine, Division of Gastroenterology and Hepatology, Chonnam National University Medical School, 42, Jaebongro, Dong-ku Gwangju, 501-757 South Korea

## Abstract

**Background:**

Eradication rate for *Helicobacter pylori* (*H. pylori*) has decreased due to antibiotic resistance. Therefore, new strategies are needed to enhance *H. pylori* eradication, especially for *H. pylori* with high antibiotic resistance. The objective of this study was to evaluate anti-*H. pylori* activities of constituents from key lime (*Citrus aurantifolia*) and their possible inhibitory effects on urease activity of *H. pylori.*

**Methods:**

*Helicobacter pylori* strain ATCC 43526 and triple drug resistant (TDR) *H. pylori* strains were used in this study. Urease activities of *H. pylori* strains were measured by ammonia colorimetrical quantification using ELISA reader. Minimum inhibitory concentrations were determined by agar dilution method for antibiotics and by modified media dilution method for each constituent of *Citrus aurantifolia* (*C. aurantifolia*).

**Results:**

*Citrus aurantifolia* extract decreased the number of colonies of *H. pylori* strain ATCC 43526 and TDR *H. pylori stains*. An increasing concentration of *C. aurantifolia* extract attenuated urease activities of *H. pylori* strain ATCC 43526 and TDR *H. pylori* strains. Among constituents of *C. aurantifolia*, citral and 4-hexen-3-one were found to be able to inhibit the growth of *H. pylori* strain ATCC 43526 and TDR *H. pylori* strains. Furthermore, citral and 4-hexen-3-one inhibited urease activities of *H. pylori* strain ATCC 43526 and TDR *H. pylori* strains in a dose-dependent manner.

**Conclusion:**

*Citrus aurantifolia* has antimicrobial effect on TDR *H. pylori* strains, suggesting that *C. aurantifolia* might have therapeutic potential to control antibiotic-resistant *H. pylori* strains that cause eradication failure using other antibiotics.

## Background

*Helicobacter pylori* (*H. pylori*) is known to be a major pathogen in the development of gastritis, peptic ulcer disease, gastric adenocarcinoma, and mucosa associated lymphoid tissue (MALT) lymphoma [1, 2]. Therefore, *H. pylori* eradication is important for the management of these diseases. However, eradication rate for *H. pylori* has decreased due to antibiotic resistance of *H. pylori*. Its resistance rates to clarithromycin and metronidazole in East Asia and Europe have been reported to be 17–34 and 28–65%, respectively [3–7]. Therefore, new alternatives or adjuvant approaches are needed for *H. pylori* eradication, especially in the area where there is high antibiotic resistance rate of *H. pylori*. Previous study has reported that foods or components of foods have anti-*H. pylori* activities by facilitating penetration of antibiotics to *H. pylori* by damaging cell membrane, inhibiting urease activity of *H. pylori*, inhibiting *H. pylori* adhesion to gastric mucosa, and interfering with cell division process of *H. pylori* [8]. Among these mechanisms, inhibiting urease activity of *H. pylori* can help eradicate *H. pylori* by altering optimal pH and inhibiting colonization of *H. pylori* [9–11].

A recent meta-analysis has shown that the intake of citrus fruits can reduce the incidence of gastric cancer in the area with high prevalence of *H. pylori* [12]. Another report has shown that phytochemical constituents of citrus peels possess biological activities, including anticancer, immunostimulation, and antigenotoxic effects [13]. Oranges, lemons, limes, grapefruit, and tangerines are well-known examples of citrus fruits. *Citrus aurantifolia* (*C. aurantifolia*), also known as key lime, is one of widely consumed citrus fruits in many cultural cuisines and juice production. It has antibacterial activities against *Mycobacterium tuberculosis, Staphylococcus aureus,* and others. Among various constituents of *C. aurantifolia*, citral, 4-Hexen-3 one, oleic acid, and palmitic acid have been found to possess antibacterial activities [14–19]. However, it is currently unclear whether *C. aurantifolia* and its constituents have anti-*H. pylori* activities. Therefore, the objective of this study was to evaluate anti-*H. pylori* activities of *C. aurantifolia* and its constituents and their possible inhibitory effects on urease activity of *H. pylori.*

## Methods

### Key lime (*C. aurantifolia*) extraction

Slices of *C. aurantifolia* were dried in a constant drying oven (VS-4150ND, VISION SCIENTIFIC, Daejeon, Korea) at temperature of 50 °C. Dried *C. aurantifolia* slices were mixed with liquid nitrogen and ground into fine powders using a mortar and pestle. Powders of *C. aurantifolia* (1 g) were then dissolved in 30 ml of sterile distilled water and incubated at room temperature for 24 h. Dissolved *C. aurantifolia* was filtered using a 0.45 µm pore syringe filter (Corning, NY 14831-001, USA). Twofold serial dilutions of *C. aurantifolia* extract (original concentration, 34 mg/ml) were made with distilled water (1:1 to 1:1024).

We used 4-hexen-3 one, oleic acid, and palmitic acid as constituents of *C. aurantifolia* to determine their antimicrobial activities and inhibitory effects on urease activity of *H. pylori* [14, 19]. For each constituent [citral (Sigma-Aldrich #W230316, USA), 4-hexen-3 one (Sigma-Aldrich #H13001, USA), oleic acid (Sigma-Aldrich #O1008, USA), and palmitic acid (Sigma-Aldrich #P0500, USA)], we prepared the following concentrations: 1, 2, 5, 10, 50, 100, 200, 400, 500, and 1000 µg/ml.

### *Helicobacter pylori* strain ATCC 43526 and triple drug resistant (TDR) *H. pylori* strains

We used standard *H. pylori* strain (ATCC^®^ CRL-43526™, USA) and TDR *H. pylori* strains isolated from gastric antrum and body from 18 patients with gastric epithelial neoplasm. Methods of isolation and culture for *H. pylori* were the same as those described in our previous study [20].

### Antimicrobial susceptibility testing

We stored *H. pylori* strains at − 80 °C. After thawing and culture of standard *H. pylori* strain and 18 TDR *H. pylori* strains, we measured minimum inhibitory concentrations (MICs) by agar dilution method for antibiotics and by modified media dilution method for *C. aurantifolia* extract and each constituent of *C. aurantifolia*. We made agar plates using Muller Hinton agar containing 5% sheep blood (Hanilcomed, Korea), 1% IsoVitalex (BD Biosciences), and one of the following drug concentrations for MIC assay: 2–32 µg/ml of metronidazole, 0.25–4 µg/ml of clarithromycin, 0.125–2 µg/ml of amoxicillin and levofloxacin, and 1–16 µg/ml of tetracycline. All antibiotics used in this investigation were purchased from Sigma (St. Louis, MO, USA) except clarithromycin which was obtained from Abbott Laboratories (Abbott Park, IL, USA). We added 10 ml of agar solution into 100 π plate and then cooled down. *H. pylori* strain ATCC 43526 (Manassas, VA USA,) was used as a quality control organism. Antibiotic concentrations used in this study were based on cutoff levels related to Laboratory Standards Institute (CLSI) clinical breakpoints for resistance. All MICs were interpreted using CLSI breakpoints. Antibiotic resistance was defined as follows: amoxicillin, MIC ≥ 0.5 µg/ml; clarithromycin, MIC > 1.0 µg/ml; metronidazole, MIC > 8 µg/ml; tetracycline, MIC > 4 µg/ml; and levofloxacin, MIC > 1 µg/ml.

We tested MIC for *C. aurantifolia* and four constituents of *C. aurantifolia*. We mixed 6 × 10^8^ CFU/ml *H. pylori* in twofold serial dilutions of *C. aurantifolia* extract (34 mg/ml–33.2 µg/ml, 1:1 to 1:1024) or in serial concentrations of its four constituents (1–1000 µg/ml), respectively. These mixtures of *H. pylori* with *C. aurantifolia* extract or its four constituents (5 µl each) were dropped immediately onto agar plates. We determined MIC levels of *C. aurantifolia* extract and each constituent based on invisible *H. pylori* colony on the agar plate after 7 days of incubation.

### Urease activity inhibition test

We harvested *H. pylori* in 0.9% saline and then prepared mixtures of 6 × 10^8^ colony forming units (CFU)/mL of *H. pylori* with two-fold serially diluted solution of *C. aurantifolia* extract (1:1 to 1:1024). We prepared 6 × 10^8^ CFU/ml of *H. pylori* with citral, 4-hexen-3-one, oleic acid, or palmitic acid in the following concentrations: 10, 50, 100, 200, 400, 500, and 1000 µg/ml*. H. pylori* strains with each constituent were incubated at room temperature for 10 min. We used 0.9% saline as a control. We added each *H. pylori* strain (6 × 10^8^ CFU/ml) in 5 into 200 µl of the following mixture: 1.5% urea (Bioshop, Canada Inc.) and 0.1% EDTA with 0.02% cresol red solution (Bioshop, Canada Inc.). The mixture ratio was 2:1. The reaction was incubated at room temperature for 20 min. After that, we measured urease activity at absorbance of 590 nm using a VersaMax™ ELISA reader (MOLECULAR DEVICES, Silicon Valley, CA, USA) [21, 22]. Urease inhibition test for each *H. pylori* strain was repeated three times.

### Statistical analysis

Analysis of variance (ANOVA) was used to determine whether there were any statistically significant differences in urease activity depending on the concentration of *C. aurantifolia* extract and its constituents. Urease activities were shown as mean ± standard deviation (SD). All reported *P* values were two-sided and *P* < 0.05 was considered statistically significant. Statistical analyses were performed using IBM SPSS software, version 23 (IBM Corp, Armonk, NY, USA).

## Results

### TDR *H. pylori* strains

According to MIC data of clarithromycin, metronidazole, and levofloxacin for *H. pylori* in our previous study [20], TDR *H. pylori* strains were all resistant to clarithromycin, metronidazole, and levofloxacin. Results are summarized in Table 1.

**Table 1:** Triple drug resistant *Helicobacter pylori* and antimicrobial activities of four components of *Citrus aurantifolia*

Strain no.	MIC (μg/ml)						
	Citral	4-Hexen-3-one	Oleic acid	Palmitic acid	Clarithromycin	Metronidazole	Levofloxacin
TDR 1	5–10	20–50	R	R	R	R	R
TDR 2	10–20	50–100	R	R	R	R	R
TDR 3	5–10	20–50	R	R	R	R	R
TDR 4	10–20	100–200	R	R	R	R	R
TDR 5	10–20	50–100	R	R	R	R	R
TDR 6	10–20	50–100	R	R	R	R	R
TDR 7	100–200	100–200	R	R	R	R	R
TDR 8	5–10	20–50	R	R	R	R	R
TDR 9	10–20	20–50	R	R	R	R	R
TDR 10	2–5	5–10	R	R	R	R	R
TDR 11	5–10	50–100	R	R	R	R	R
TDR 12	2–5	20–50	R	R	R	R	R
TDR 13	10–20	20–50	R	R	R	R	R
TDR 14	5–10	20–50	R	R	R	R	R
TDR 15	10–20	20–50	R	R	R	R	R
TDR 16	5–10	20–50	R	R	R	R	R
TDR 17	2–5	20–50	R	R	R	R	R
TDR 18	2–5	20–50	R	R	R	R	R
ATCC 43526	2–5	20–50	R	R	S	R	S

*No.* number, *MIC* minimum inhibitory concentration, *TDR* triple drug resistant, *R* resistant, *S* sensitive, *ATCC 43526 Helicobacter pylori* strains ATCC 43526

### Effect of *C. aurantifolia* extract on growth and urease activities of *H. pylori* strain ATCC 43526 and TDR *H. pylori* strains

**Fig. 1:**
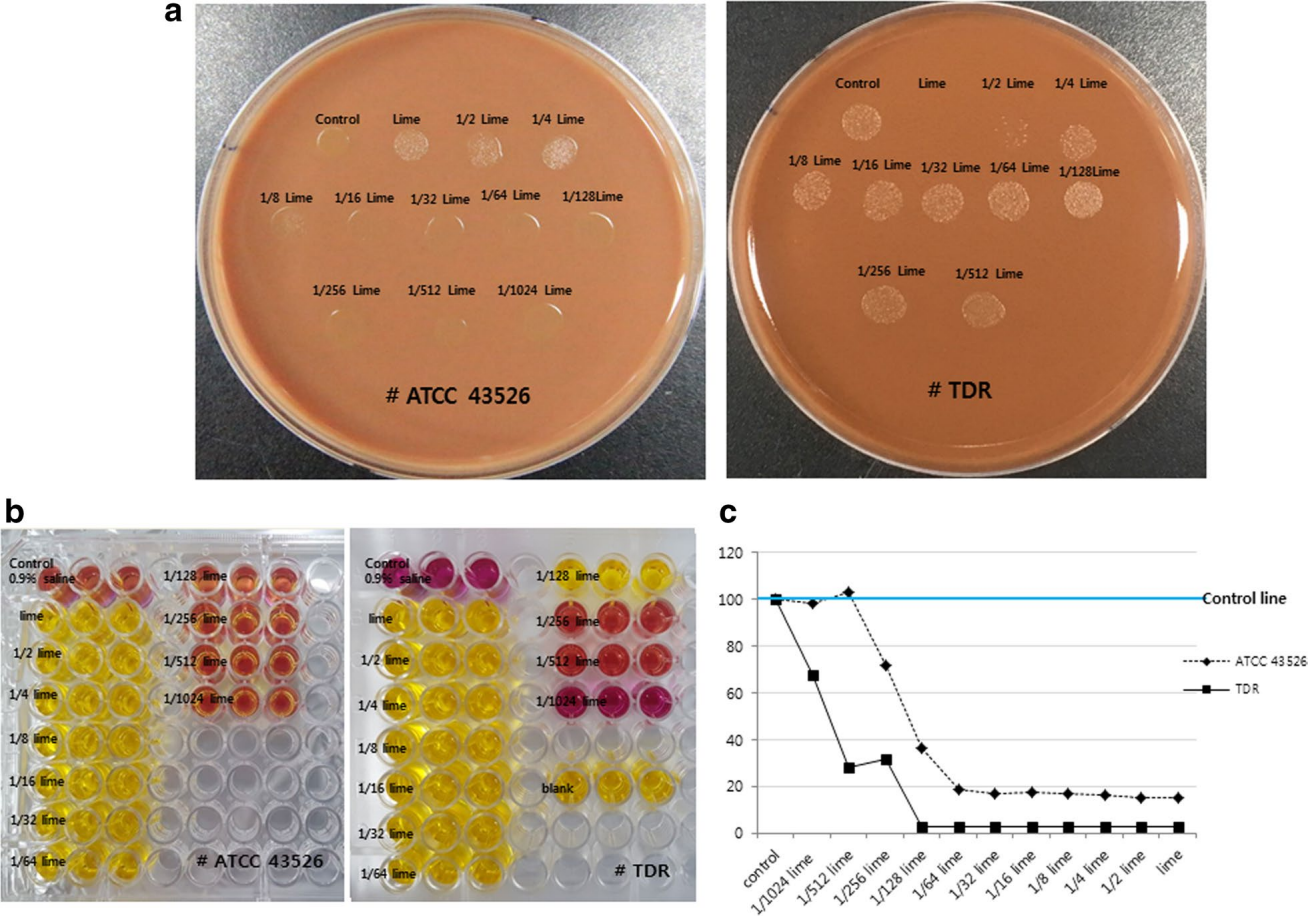
**a** Effect of *C. aurantifolia* extract on growth of standard *H. pylori* strain ATCC 43526 and triple drug resistant (TDR) *H. pylori* strain. The number of visible colonies of *H. pylor*i strain ATCC 43526 or TDR *H. pylori* was decreased in the presence of *C. aurantifolia* extract compared to that in the control without the presence of *C. aurantifolia* extract. **b** Urease activity test of standard *H. pylori* strain ATCC 43526 and triple drug resistant (TDR) *H. pylori* strain. We determined inhibitory effects of *C. aurantifolia* extract at dilution of 1:1 to 1:64 on urease activity of *H. pylori* strain ATCC 43526 and at dilution of 1:1 to 1:128 on urease activity of TDR *H. pylori.* **c** Dose-dependent inhibitory effect of *C. aurantifolia* extract on urease activities of *H. pylori* strain ATCC 43526 and triple drug resistant *H. pylori* strain. An increasing concentration of *C. aurantifolia* extract showed higher attenuation of urease activity (*P* < 0.001)

First, we evaluated the effect of *C. aurantifolia* extract on the growth of standard *H. pylori* strain ATCC 43526 and TDR *H. pylori* strains. We observed visible growth of *H. pylori* mixed with *C. aurantifolia* after twofold serial dilution (1:1 to 1:1024) on agar plate after 7 days of inoculation. The number of visible colonies of *H. pylori* strain ATCC 43526 and TDR *H. pylori* strains was decreased in the presence of *C. aurantifolia* extract compared to that in the control without the presence of *C. aurantifolia* extract (Fig. 1a). We measured urease activities of *H. pylori* strain ATCC 43526 and TDR *H. pylori* strains mixed with *C. aurantifolia* extract at each dilution. Results for their inhibitory effects on urease activities of *H. pylori* strain ATCC 43526 and TDR *H. pylori* strains at each concentration of *C. aurantifolia* extract are shown in Fig. 1b. With increasing concentration of *C. aurantifolia* extract, higher attenuation of urease activity of *H. pylori* was observed (*P *< 0.001, Fig. 1c, Table 2). *H. pylori strains* ATCC 43526 treated with *C. aurantifolia* extract at dilution of 1:64 showed 18.77 ± 1.74% of urease activity compared to that of the control whereas TDR *H. pylori* strains treated with *C. aurantifolia* extract at dilution of 1:128 showed 2.62 ± 0.05% of urease activity compared to that of the control (Table 2).

**Table 2:** Inhibition effect of *Citrus aurantifolia* extract on urease activities of *Helicobacter pylori* strains ATCC 43526 and triple drug resistant *Helicobacter pylori*

Dilution titer of *citrus aurantifolia*	ATCC 43526 Urease activity (%)	TDR Urease activity (%)
Control	100	100
1/1024	98.3	67.6
1/512	103.0	28.0
1/256	71.8	31.5
1/128	36.6	2.6
1/64	18.8	2.7
1/32	17.0	2.7
1/16	17.6	2.6
1/8	16.9	2.6
1/4	16.1	2.7
1/2	15.2	2.7
1	14.9	2.8

*ATCC 43526 Helicobacter pylori* strains ATCC 43526, *TDR* triple drug resistant

### Effect of citral, 4-hexen-3-one, oleic acid, and palmitic acid on growth and urease activities of *H. pylori* strain ATCC 43526 and TDR *H. pylori* strains

**Fig. 2:**
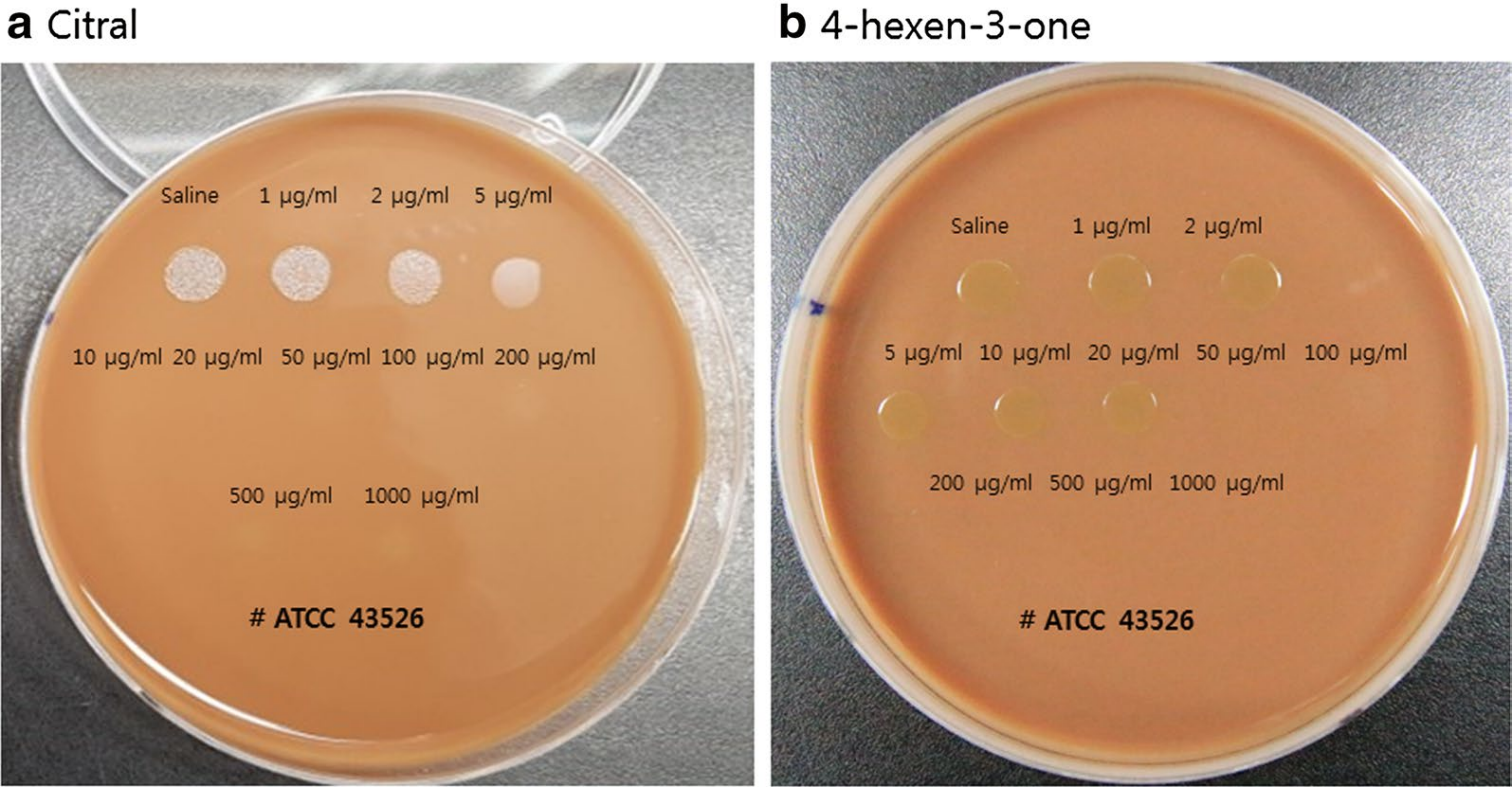
Effect of citral (**a**) and 4-hexen-3-one (**b**) on the growth of standard *H. pylori* strain ATCC 43526. Small spot without convexity and translucency is only trace of drop. Citral had inhibitory effect on standard *H. pylori* strain ATCC 43526, with MIC of 2–5 μg/ml. 4-Hexen-3-one also had inhibitory effect on standard *H. pylori* strain, with MIC of 20–50 μg/ml

We evaluated effects of constituents from *C. aurantifolia* on the growth of standard *H. pylori* strain ATCC 43526. We found visible growth of *H. pylori* colony treated with low dose of citral (≤ 2 μg/ml) and low dose of 4-hexene-3-one (≤ 20 μg/ml) on agar plate after 7 days of inoculation. Citral above concentration of 5 μg/ml persistently stopped the growth of *H. pylori* (MIC, 2–5 μg/ml, Fig. 2a). 4-hexen-3-one above concentration of 50 μg/ml persistently stopped the growth of *H. pylori* (MIC, 20–50 μg/ml, Fig. 2b). However, oleic acid or palmitic acid showed no effect on the growth of *H. pylori* strain ATCC 43526.

**Fig. 3:**
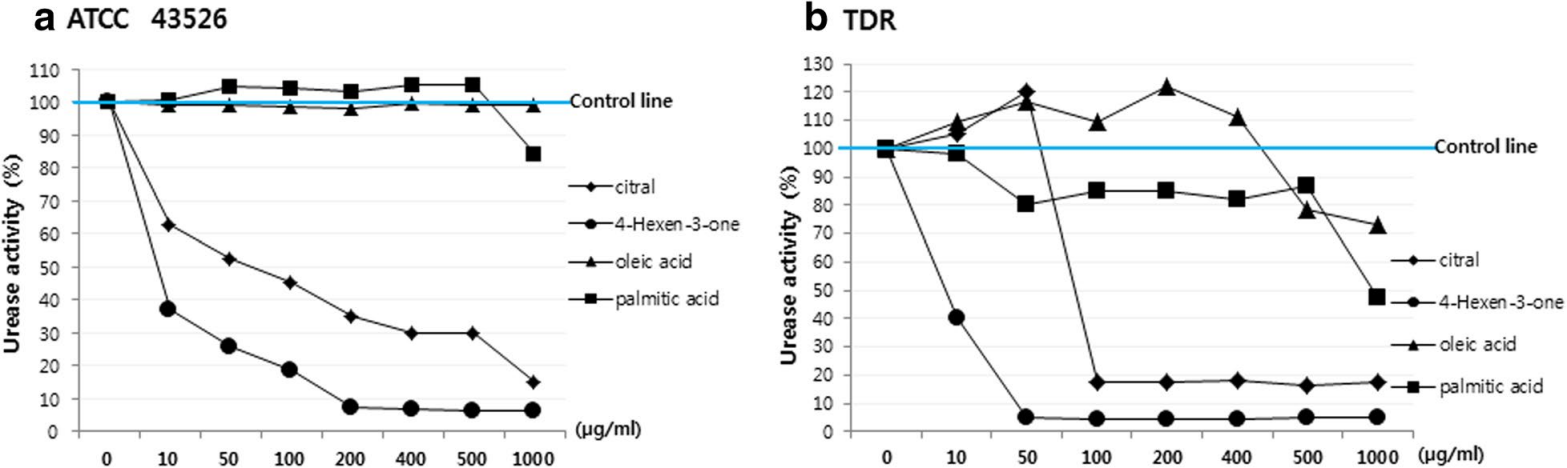
Effect of citral, 4-hexen-3-one, oleic acid, and palmitic acid on urease activities of standard *H. pylori* strain ATCC 43526 (**a**) and triple drug resistant (TDR) *H. pylori* strain (**b**). An increasing concentration of citral and 4-hexen-3-one attenuated more urease activities of *H. pylori strain* ATCC 43526 and TDR *H. pylori* strain (both *P *<0.001). However, palmitic acid and oleic acid showed no inhibition effects on urease activity (both *P* > 0.05)

We measured effects of citral, 4-hexen-3-one, oleic acid, and palmitic acid on urease activities of standard *H. pylori* strain ATCC 43526. With increasing concentration, 4-hexen-3-one showed higher attenuation effects on urease activity of *H. pylori strain* ATCC 43526 (*P *<0.001). *H. pylori* strain ATCC 43526 treated with 4-hexen-3-one at concentration of 10 µg/ml had urease activity of 37.11% compared to the control (*P *=0.006, Fig. 3a, Table 3). With increasing concentration of citral, higher attenuation of urease activity of *H. pylori strain* ATCC 43526 was achieved (*P *<0.001). *H. pylori* strain ATCC 43526 treated with citral showed decreased urease activity depending on the concentration used (*P *<0.001). *H. pylori* strain ATCC 43526 treated with citral at 100 µg/ml showed urease activity of 52.67% compared to the control (*P *=0.002, Fig. 3a, Table 3). However, palmitic acid or oleic acid showed no inhibitory effect on urease activity of *H. pylori* strain 43526 (Fig. 3a, Table 3).

**Table 3:** Inhibition effect of citral, 4-hexen-3-one, oleic acid, and palmitic acid on urease activities of *Helicobacter pylori* strain ATCC 43526 and triple drug resistant *Helicobacter pylori*

Concentration (μg/ml)	Citral Urease activity (%)		4-Hexen-3-one Urease activity (%)		Oleic acid Urease activity (%)		Palmitic acid Urease activity (%)	
	ATCC 43526	TDR	ATCC 43526	TDR	ATCC 43526	TDR	ATCC 43526	TDR
0	100	100	100	100	100	100	100	100
10	62.48	105.09	37.11	40.48	99.29	109.25	100.83	98.38
50	52.67	120.07	25.74	4.97	99.27	116.37	104.65	80.02
100	45.04	17.74	18.47	4.27	98.58	109.69	104.51	85.17
200	35.23	17.29	7.35	4.44	98.08	121.82	103.16	84.76
400	29.70	17.86	6.67	4.45	99.61	111.19	105.19	81.76
500	29.65	16.39	6.53	4.92	99.30	78.19	105.11	87.00
1000	14.75	17.26	6.38	5.14	98.90	72.97	84.51	47.34

*ATCC 43526 Helicobacter pylori* strains ATCC 43526, *TDR* triple drug resistant

Antimicrobial activities of four constituents from *C. aurantifolia* against TDR *H. pylori* strains are shown in Table 1. Oleic acid or palmitic acid showed no antimicrobial effect on TDR *H. pylori* strains. However, citral and 4-hexen-3-one inhibited the growth of TDR *H. pylori* strains.

We measured effects of citral, 4-hexen-3-one, oleic acid, and palmitic acid on urease activities of TDR *H. pylori* strains. With increasing concentration, 4-hexen-3-one showed higher attenuation effects on urease activity of TDR *H. pylori* strains (*P *<0.001). TDR *H. pylori* strains treated with 4-hexen-3-one at concentration of 10 µg/ml had urease activity of 40.48% compared to the control (*P *=0.007, Fig. 3b, Table 3). With increasing concentration of citral, higher attenuation of urease activity of TDR *H. pylori* strains was achieved (*P *< 0.001). TDR *H. pylori* strains treated with citral showed decreased urease activity depending on the concentration used (*P *< 0.001). TDR *H. pylori* strains treated with citral at concentration of 100 µg/ml showed urease activity of 17.74% compared to the control (*P *< 0.001, Fig. 3b, Table 3). However, palmitic acid or oleic acid showed no inhibitory effect on urease activities of *TDR H. pylori* strains (Fig. 3b, Table 3).

## Discussion

Our present study showed that *C. aurantifolia* extracts could inhibit urease activity of antibiotic-susceptible *H. pylori* strain and TDR *H. pylori* strains in vitro in a dose-dependent manner. Among constituents of *C. aurantifolia,* citral and 4-hexen-3-one showed dose-dependent inhibition of urease activities of antibiotic-susceptible *H. pylori* strain and TDR *H. pylori* strains. Furthermore, citral and 4-hexen-3-one showed inhibitory effects on the growth of antibiotic-susceptible *H. pylori* strain and TDR *H. pylori* strains.

*Helicobacter pylori* eradication rates have decreased while their resistance rates to antibiotics have increased. To improve eradication rates of *H. pylori*, alternative treatments such as antibiotics combined with plant extracts, probiotics, curcumin, honey, and antioxidants have been suggested [8]. Previous study has shown that lime juice concentrates have good inhibitory effects on both Gram-negative and Gram-positive bacterial strains, with MIC in the range of 12.5–50 μg/ml [23]. Another study has demonstrated that hexane extract of fruit peels of *C. aurantifolia* exhibits inhibitory effect against antimicrobial resistant *M. tuberculosis* strains, with MIC in the range of 25–50 μg/ml [24]. Among constituents from *C. aurantifolia,* palmitic acid, linoleic acid, oleic acid, 4-hexen-3-one, and citral are active against *M. tuberculosis* strains [14, 25]. We selected four available constituents (palmitic acid, oleic acid, 4-hexen-3-one, and citral) from *C. aurantifolia* that showed antimycobacterial activity. In our study, *C. aurantifolia* extract decreased the number of *H. pylori* ATCC 43526 colonies and TDR *H. pylori* colonies. Constituents of *C. aurantifolia* also showed inhibitory effects against *H. pylori* strain ATCC 43526, with MIC of citral at 5–10 μg/ml and MIC of 4-hexen-3-one at 20–50 μg/ml. Furthermore, citral showed inhibitory effects against 18 *H. pylori* strains with triple drug resistance. Its MIC ranged from 2 to 100 μg/ml. In addition, 4-hexen-3-one showed inhibitory effects against 18 *H. pylori* strains with triple drug resistance. Its MIC was in the range of 20–200 μg/ml.

Biglar et al. have shown that *C. aurantifolia* can inhibit the activity of Jack-bean urease (IC_50_ = 28 μg/ml) [26]. Our study also showed that *C. aurantifolia* extract could inhibit urease activity of *H. pylori* at dilution of 1:64 to 1:1. Among constituents from *C. aurantifolia*, citral and 4-hexen-3-one showed dose-dependent inhibitory effects on urease activity of *H. pylori*. It is known that *H. pylori* can neutralize acid in its environment by producing urease which breaks down urea in the stomach to carbon dioxide and ammonia. These chemicals then react with strong acids in the gastric environment to produce a neutralized area around *H. pylori* [27]. Previous animal study has shown that *H. pylori* is unable to colonize at gastric mucosa with normal physiological pH in urease-negative mutant piglet [28]. Recently, another study has demonstrated that bacterial load is decreased within 5–7 days in a urease knockout infection mouse model [29]. Urease expression is required for establishing initial colonization and maintaining chronic infection [2, 29]. In the present study, *C. aurantifolia* extract and its constituents showed inhibitory effects on urease activity of *H. pylori*, suggesting that they might have potential as adjuvants to enhance *H. pylori* eradication.

In this study, we did not show the association between antibacterial effect and inhibition of urease activity. Bactericidal effect of *C. aurantifolia* may affect the growth of *H. pylori* colonies, leading to inhibition of urease activity and vice versa. Although low dose of *C. aurantifolia* extract showed no obvious effect on the growth of *H. pylori*, it showed inhibitory effect on urease activity of *H. pylori*. Further studies are needed to evaluate the mechanism involved in the antibacterial effect of *C. aurantifolia* and the causal association between its inhibition of urease activity and bactericidal effects.

In conclusion, *C. aurantifolia* and its constituents attenuated urease activities of *H. pylori* strains. Citral and 4-hexen-3-one had antimicrobial effects on *H. pylori* strains with triple drug resistance, suggesting that *C. aurantifolia* might have potential as a therapeutic agent to control *H. pylori* strains that cause eradication failure with other antibiotics. Future studies are needed to evaluate the efficacy and toxicity of *C. aurantifolia* in vivo.
